# Co-Transfected Plasmids Enhance Transient Expression of Reporter Genes

**DOI:** 10.3390/biotech15010023

**Published:** 2026-03-04

**Authors:** Shih-Yen Lo, Chee-Hing Yang, Yu-Ru Chan, Yi-Tzu Chao, Meng-Jiun Lai, Hui-Chun Li

**Affiliations:** 1 Department of Laboratory Medicine and Biotechnology, Tzu Chi University, Hualien 970, Taiwan; losylo@gms.tcu.edu.tw (S.-Y.L.); microbiology721@gms.tcu.edu.tw (Y.-R.C.); 114202102@gms.tcu.edu.tw (Y.-T.C.); monjou@mail2.tcu.edu.tw (M.-J.L.); 2 Department of Laboratory Medicine, Buddhist Tzu Chi General Hospital, Hualien 970, Taiwan; 3 Department of Microbiology and Immunology, School of Medicine, Tzu Chi University, Hualien 970, Taiwan; cheehing2@gms.tcu.edu.tw; 4 Department of Biochemistry, School of Medicine, Tzu Chi University, Hualien 970, Taiwan

**Keywords:** transient transfection, co-transfection, reporter genes, enhanced gene expression, histones, Firefly luciferase, Renilla luciferase

## Abstract

Transient DNA transfection is routinely used to study gene function and elucidate the regulation of biological pathways, and it is also widely applied in biotechnology for large-scale recombinant protein production. The results of recent studies involving mammalian cells have highlighted that competition for cellular resources during gene expression can bias data interpretation, directly affecting co-transfection experiments. In this study, our results showed that co-transfected plasmids markedly enhance transient—but not stable—expression of various reporter genes across different cell types. The enhancement of transient reporter gene expression by additional plasmid DNA occurs when these DNAs are co-delivered simultaneously and is unlikely to be mediated by cytokine induction. Furthermore, co-transfected plasmids were shown to upregulate transcription, but not translation, of the reporter gene during transient expression. Thus, the observed enhancement may result from competition between co-transfected plasmids and reporter constructs for cellular proteins that interact with transfected DNA, such as histones. Indeed, Pracinostat (SB939), an inhibitor of histone deacetylase, was able to enhance the transient expression of the reporter gene dose-dependently. Overall, this study provides insights that may facilitate improved transient expression of recombinant genes in biotechnological applications.

## 1. Introduction

A wide range of recombinant proteins, such as monoclonal antibodies, have been applied to the treatment of various diseases. Indeed, a substantial proportion of biopharmaceuticals approved by the U.S. Food and Drug Administration (FDA) consist of recombinant monoclonal antibodies (rMAbs) or their conjugates. Chinese hamster ovary (CHO) cells have become the predominant host system for rMAb production owing to their robustness, ease of transfection, and ability to perform post-translational modifications similar to those of human cells [1]. Expression of rMAbs in CHO cells can be achieved through either transient transfection or stable integration. Transient gene expression systems in mammalian cells have gained popularity for their capacity to rapidly and efficiently produce large quantities of recombinant proteins. These systems also offer scalability without the time-consuming steps required to establish stable cell lines. To further enhance rMAb yield in CHO cells, numerous strategies have been developed, including vector optimization, medium formulation, cultivation parameter adjustment, and cell engineering [2].

Transient transfection is also widely used in basic and synthetic biology to elucidate the regulation of biological pathways. When plasmids are introduced into mammalian cells, they are transcribed and translated to produce the desired proteins [3]. However, transiently expressed genes compete for limited transcriptional and translational resources, coupling otherwise independent exogenous and endogenous genes and creating discrepancies between intended and actual gene function. The expression level of a transfected plasmid depends on the strength of its promoter and other regulatory elements. Efficient transient expression of foreign genes in mammalian cells depends on the successful delivery, cytoplasmic transport, and nuclear import of the introduced vector [4,5].

Reporter genes such as luciferase and green fluorescent protein (GFP) are frequently employed to monitor gene expression due to their ease of detection [6,7]. Reporter constructs are widely employed to study eukaryotic gene regulation and expression. Due to the inherent variability in experiments, reporter gene expression is typically normalized with co-delivered genes that act as transfection controls. However, the results of recent studies have demonstrated that competition for cellular resources during gene expression can bias data interpretation, particularly in co-transfection experiments [8,9], with the authors of these previous studies focusing on the interactions between two expressing plasmids [8,9]. In this study, we intend to investigate the effects of co-transfected empty vectors on reporter gene expression. Our hypothesis is that the co-transfected empty vectors could also modulate the transient gene expression of the reporters. It is our desire to determine whether the enhancement of transient reporter expression by the co-transfected empty vectors is promoter-dependent, reporter-sequence-dependent, or cell-type-dependent. Furthermore, we intend to unveil the underlying mechanism(s) by distinguishing between transcription vs. translation and linking these processes to chromatin/histone-related mechanisms.

In this study, we found that co-transfected plasmids dramatically enhance transient—but not stable—expression of different reporter genes across various cell types. This enhancement may be consistent with competition between co-transfected plasmids and reporter constructs for cellular proteins that interact with transfected DNA, such as histones. Inhibition of these proteins further increases transient reporter gene expression. Overall, this study provides insights that could facilitate more efficient transient expression of recombinant genes for biotechnological applications.

## 2. Materials and Methods

### 2.1. Plasmid Construction and DNA Transfection

The co-transfected plasmids used in this study are commercially available: pcDNA3.1-V5-HisA (https://www.novoprolabs.com/vector/V11329, accessed on 25 November 2025), PUC19 (https://www.addgene.org/50005/, accessed on 25 November 2025), pCAG vector (https://rnai.genmed.sinica.edu.tw/file/vector/, accessed on 25 November 2025), pBlueScript (https://bioactiva.com/en/pbluescript-ii-ks, accessed on 25 November 2025), pBD-GAL4-Cam and pAD-GAL4 (www.genomics.agilent.com, accessed on 25 November 2025). Some of the reporters used in this study are also commercially available: pGL3Basic (https://www.addgene.org/212936, accessed on 25 November 2025), pEGFP-C1 (https://www.addgene.org/vector-database/2487/, accessed on 25 November 2025) and pRL-TK (https://worldwide.promega.com/products/luciferase-assays/, accessed on 25 November 2025). The plasmid v9-luc with an inducible promoter was kindly donated by Dr. King-Song Jeng (https://rnai.genmed.sinica.edu.tw/file/vector/, accessed on 25 November 2025). Some expression plasmids used as reporters in this study were constructed using different vectors: pLAS2w-pPuro, pLAS3w-pPuro, pLAS5w-pPuro, pCAG vector (https://rnai.genmed.sinica.edu.tw/file/vector/, accessed on 25 November 2025), and pcDNA4 vector (https://www.thermofisher.com, accessed on 25 November 2025). Inserts of DNA fragments were amplified by means of polymerase chain reaction (PCR) with Phusion™ High-Fidelity DNA Polymerase (Thermo Scientific, Waltham, MA, USA). The primer sequences used for PCR are available upon request. Following PCR amplification, DNA fragments were cloned into expressing vectors via digestion with restriction enzymes and ligation with T4 DNA ligase (Takara Bio, San Jose, CA, USA). The characteristics of these reporters are described in Table 1. pISRE-Luc, kindly donated by Prof. R.L. Kuo (Research Center for Emerging Viral Infections, College of Medicine, Chang Gung University, Taoyuan, Taiwan), is a luciferase reporter plasmid under the control of the interferon-stimulated response element (ISRE) promoter [10]. All plasmids used in this study were verified by means of sequencing. The polyethylenimine (PEI, linear, MW 25,000) used to transfect DNA into cells was purchased from Polysciences Inc. (Warrington, PA, USA).

### 2.2. Cell Culture

The cell lines used in this study, including A549, Madin–Darby canine kidney (MDCK), 293T, Vero, Huh7, and HeLa cells, have been described previously [15,16]. A549, MDCK, 293T, and HeLa cells were obtained from Dr. King-Song Jeng (http://rnai.genmed.sinica.edu.tw). A549 cells were cultured in Ham’s F12 Nutrient Mixture with Kaighn’s Modification (F12K, Sigma, St. Louis, MO, USA), containing 2.5 g/L NaHCO_3_, 10% fetal bovine serum (FBS), 100 U/mL penicillin, and 100 μg/mL streptomycin (P + S) (Gibco, Waltham, MA, USA). MDCK, 293T cells, and Vero and Huh7 cells were cultured in Dulbecco’s modified Eagle’s medium (DMEM) containing 10% FBS, 1% glutamine, 1 mM sodium pyruvate, and P + S. HeLa cells were cultured in RPMI medium 1640 (Gibco) containing 2 g/L NaHCO_3_, 10% FBS, and P + S. All cultured cells were maintained at 37 °C with 5% CO_2_. HeLa (WT) and HeLa (eIF2aK2KO) cells were purchased from a biotech company (https://www.abcam.com/). HeLa cells stably transfected with H2B-GFP were provided by Noriaki Shimizu (Graduate School of Integrated Sciences for Life, Hiroshima University, Japan) [6,17]. All cultured cells were maintained at 37 °C with 5% CO_2_. To determine whether plasmid co-transfection consistently enhances reporter gene expression across different biological backgrounds, we repeated the experiment using a diverse range of cell lines.

### 2.3. RNA Transfection

A total of 0.2 μg of commercially available CleanCap^®^ Luc mRNA (https://shop.trilinkbiotech.com) was transfected into cells using TransMessenger Transfection Reagent (https://www.qiagen.com/us/products/, accessed on 25 November 2025) following the manufacturer’s instructions. At 24 h after transfection, luciferase activity was measured.

### 2.4. Western Blotting Analysis

Protein lysates for SDS-PAGE were collected from 7 × 10^5^ HeLa (WT) and HeLa (eIF2aK2KO) cells. Following electrophoresis, total proteins from the SDS-PAGE gel were transferred to a 0.2 μm PVDF membrane (Pall Corporation, New York, NY, USA). All procedures were then performed at room temperature [15,18]. The primary antibodies used were antibodies against PKR (Santa Cruz Biotechnology, Dallas, TX, USA) and Beta-Actin (Genetex, Irvine, CA, USA). In this assay, β-actin was used as the loading control. Thereafter, the membrane was washed three times for 10 min with 1 × PBST and hybridized with the individual secondary antibodies at 1:4000 dilution in 1 × PBST for 1 h at room temperature. The secondary antibodies used were goat anti-rabbit IgG (HRP), goat anti-mouse IgG (HRP), or goat anti-rabbit IgG (AP). The PVDF membrane was subsequently washed three times with 1 × PBST, and enhanced chemiluminescence (ECL) reagent (Thermo Fisher Scientific, Waltham, MA, USA) was applied. Autography was obtained via X-ray film or UVP BioSpectrum 810 (Thermo Fisher Scientific, Waltham, MA, USA).

### 2.5. Stable Cell Establishment

Stable cell establishment was conducted with the lentiviral expressing system (http://rnai.genmed.sinica.edu.tw), following the manufacturer’s instructions and our previous procedures [7]. In brief, pseudo-typed lentiviruses were generated by co-transfecting HEK-293T cells with plasmid DNA mixtures of pCMVΔ8.91, pMD.G, and the plasmid for the reporter gene (e.g., pLAS5w-Fluc, IFN-Fluc, ISRE-Fluc) using a PEI transfection reagent. The pseudo-typed lentiviruses were harvested at 48 h post transfection. After harvest, the lentiviruses were filtered through a 0.45 μm filter and used for transduction with 8 μg/mL polybrene, followed by puromycin (2 μg/mL) selection in HeLa cells.

### 2.6. Luciferase Assay

A total of 1 × 10^5^ cells of various types, including HeLa, Huh7, A549, and Vero, were co-transfected with various reporters (0.1 μg pcDNA3-Fluc, 0.1 μg pcDNA3-Rluc, or 0.05 pRL-TK) plus different doses of plasmids (e.g., 0.1 μg, 0.2 μg and 0.4 μg of pUC19) in the presence of the same amount of PEI (3 μg). Cells were harvested 48 h after transfection. The dual-luciferase assay system (Promega, Madison, WI, USA) was used following the manufacturer’s instructions and our previous procedures [7]. In each experiment, samples were analyzed in at least triplicate.

### 2.7. RNA Extraction and Real-Time RT-PCR

A total of 7 × 10^5^ HeLa cells were co-transfected with 1 μg of pcDNA4-Rluc plus different doses of pUC19 (0 μg, 0.5 μg, 1.5 μg, and 2.5 μg) in the presence of PEI (PEI:DNA = 6:1 in μg). Total RNAs, extracted from the cells 48 h after transfection using TRIzol reagent (Invitrogen, Thermo Fisher Scientific) based on the manufacturer’s instructions, were treated with DNase I and converted into cDNAs using oligo (dT) for the mRNA analysis as the primers [18]. A high-capacity cDNA reverse transcription kit (Applied Biosystems, ThermoFisher Scientific, Waltham, MA, USA) was used for reverse transcription. A LabStar SYBR qPCR kit (TAIGEN Bioscience Corporation, Taipei City, Taiwan) was used for the real-time PCR. The primers used for the real-time RT-PCR were QRluc-F (5′-CGTGCAAGTGATGATTTACC-3′) and QRluc-R (5′-TCTTGCGAAAAATGAAGACC-3′). In these reactions, beta-actin mRNA was used as the internal control using primers Qbetaactin-F (5′-CATCGAGCACGGCATCGTCA-3′) and Qbetaactin-R (5′-TAGCACAGCCTGGATAGCAAC-3′). All experiments were repeated at least three times, and the real-time RT-PCR procedures followed MIQE guidelines [19].

### 2.8. Statistical Analysis

Experiments involving luciferase assays were performed at least three times in triplicate. Luciferase data were normalized to the mock control, which was set to 1, and are presented as box-and-whisker plots (minimum to maximum). Data distribution was assessed using the Shapiro–Wilk normality test. Based on the overall distribution characteristics and the experimental design, two-tailed one-sample t-tests were used to determine whether each experimental group differed from the baseline reference value. When multiple comparisons were conducted, Bonferroni correction was applied, and statistical significance was indicated only when *p* values were below the adjusted threshold (ns, no significance; *p* < 0.05, *; *p* < 0.01, **; *p* < 0.001, ***; *p* < 0.0001, ****). Statistical analyses were performed using GraphPad Prism version 10 (GraphPad Software, San Diego, CA, USA).

## 3. Results

Co-transfected plasmids enhance transient but not stable expression of the reporter in various cell types.

**Figure 1:**
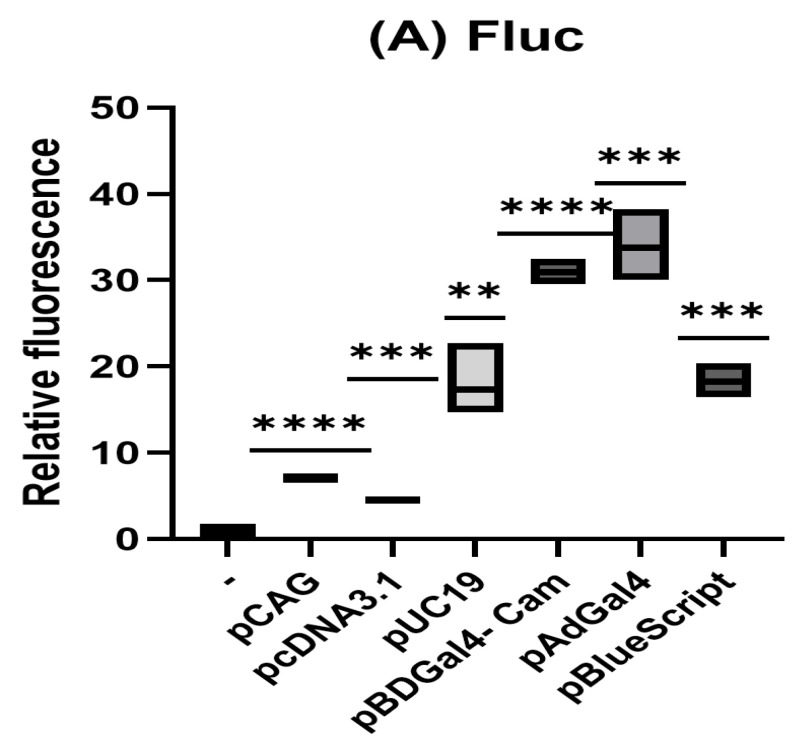

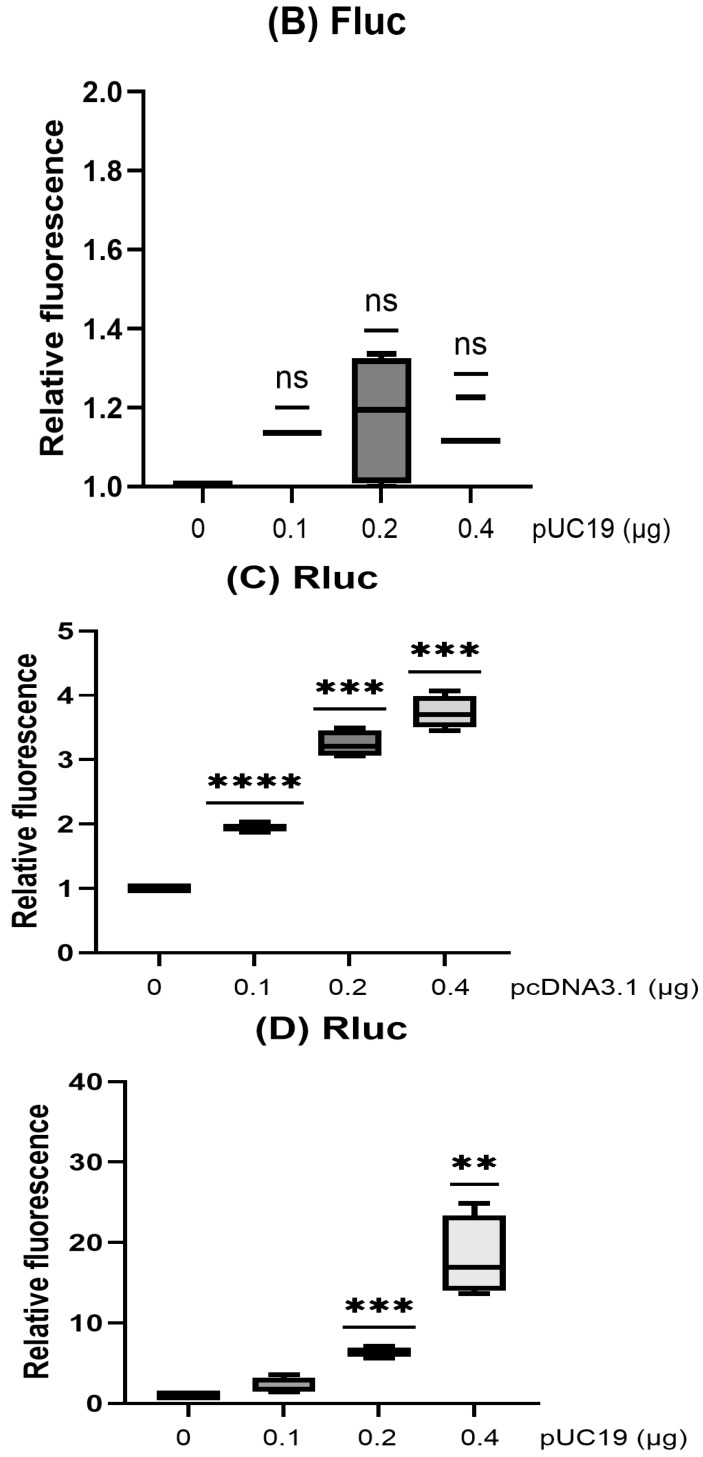

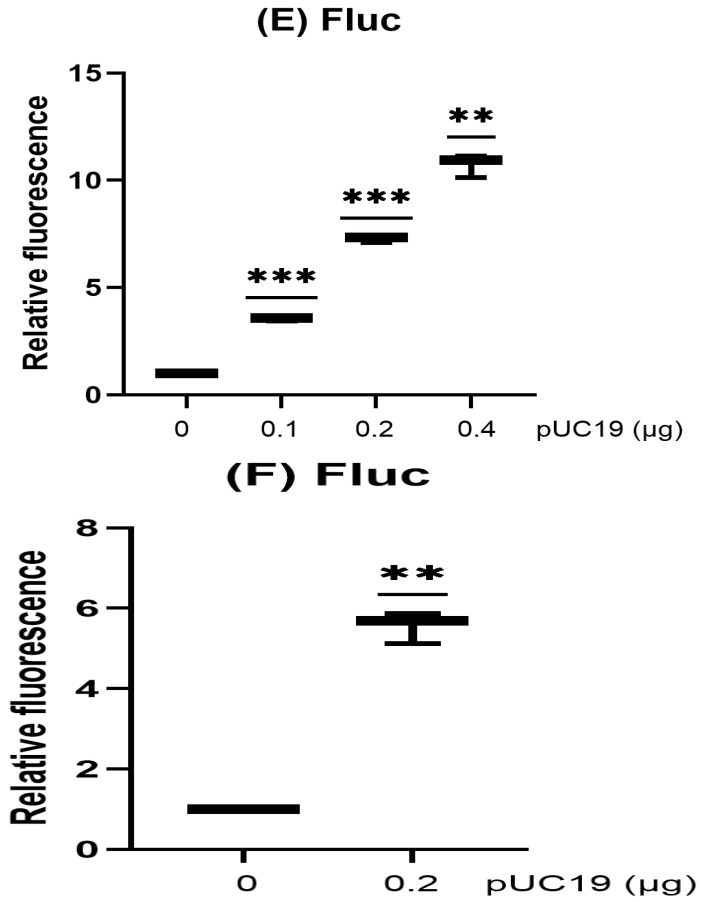
Experiments were performed in triplicate. Luciferase data were normalized to the mock control, which was set to 1. (**A**) HeLa cells were co-transfected with pLas5w-Fluc 0.1 μg and either mock-transfected or transfected with various plasmids at 0.4 μg as indicated in the same amount of PEI reagent. Luciferase activity was analyzed 48 h after transfection. (**B**) HeLa cells stably transfected with pLas5w-Fluc were transfected with different amounts of PUC19 plasmid as indicated in the same amount of PEI reagent. Luciferase activity was analyzed 48 h after transfection. (**C**). HeLa cells were co-transfected with pcDNA4-Rluc 0.1 μg and different amounts of pcDNA3.1-V5-His plasmid as indicated in the same amount of PEI reagent. Luciferase activity was analyzed 48 h after transfection. (**D**). HeLa cells were co-transfected with pcDNA4-Rluc 0.1 μg and different amounts of pUC19 plasmid as indicated in the same amount of PEI reagent. Luciferase activity was analyzed 48 h after transfection. (**E**). HeLa cells were co-transfected with 0.1 μg pGL3-SERPINE1-p700 and different amounts of pUC19 plasmid as indicated in the same amount of PEI reagent. Luciferase activity was analyzed 48 h after transfection. (**F**). HeLa cells were transfected with 0.1 μg v9-luc with or without 0.2 μg pUC19. After transfection, doxycycline (1 μg/mL) was added to induce the expression of Fluc. Luciferase activity was analyzed 48 h after transfection. (ns, no significance; *p* < 0.01, **; *p* < 0.001, ***; *p* < 0.0001, ****).

To study the effect of co-delivered plasmids on the transient expression of the reporter, various plasmids were co-transfected with the reporter (pLAS5w-Fluc) into HeLa cells. All of the co-transfected plasmids enhanced the transient expression of the reporter gene to different degrees (Figure 1A). The enhancement of the transient expression of the reporter by the co-transfected plasmids was also detected using various reporters under the control of different promoters, such as pRL-TK, pcDNA4-Fluc, pcDNA4-Rluc, pDNA4-EMCV-Rluc, pDNA4-HCVires-Rluc, pDNA4-CRPV-Rluc, both ORFs in pFr-crpv-xb, pcdna4-fluc-emcvires-rluc, pLAS2w-fluc-hcvires-rluc, and pLAS2w-fluc-crpvigr-rluc Moreover, the enhancement of the transient expression of the reporter by the co-transfected plasmids was also demonstrated in various cell lines, such as HuH7, 293T, HeLa (WT), HeLa (eIf2aK2KO), Vero, and A549 cells. Thus, enhancing transient gene expression of the reporters by the co-transfected plasmids is not promoter-dependent, not reporter-gene-sequence-dependent, and not cell-type-dependent. On the contrary, co-transfected plasmids (e.g., pUC19) did not affect the stable expression of the same reporter (pLAS5w-Fluc) when the reporter was integrated into the chromosome through a lentiviral vector (Figure 1B), and neither did the co-transfected plasmid (e.g., pUC19) enhance the stably expressed H2B-GFP in HeLa cells [6].

The results of previous studies have demonstrated that co-delivered two plasmids with the same promoter would result in interference, predominantly inhibition, between promoters [9,20,21]. Thus, we hypothesized that the co-transfected plasmids with the same promoter as that of the reporter would enhance the transient expression of the reporter less than those without the same promoter. To test this hypothesis, pcDNA3.1-V5-HA containing the CMV promoter (Figure 1C) or pUC19 (Figure 1D) was separately co-transfected with the reporter pcDNA4-Rluc with the CMV promoter. The expression enhancement of the reporter by pUC19 being significantly greater than that by pcDNA3.1-V5-HA is likely due to the absence of the CMV promoter in the pUC19 plasmid.

To avoid the promoter effect, pUC19 was thus used for this study. The co-transfected pUC19 plasmid could also enhance the transient expression of the reporter under the control of the cellular SERPINE1 gene promoter in HeLa (Figure 1E) and Huh7 cells. Moreover, the co-transfected pUC19 plasmid could also enhance inducible transient expression similar to that of transient expression (Figure 1F).

### 3.1. Co-Transfected Plasmid DNA Enhances Transient Expression Independently of Cytokine Induction

**Figure 2:**
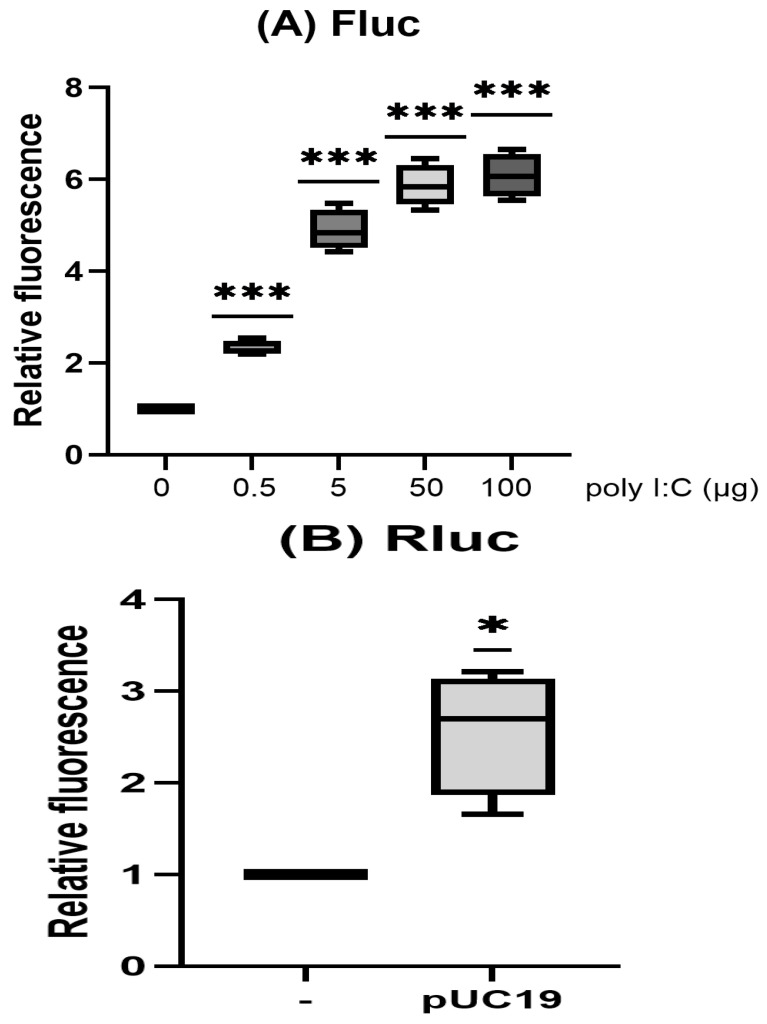

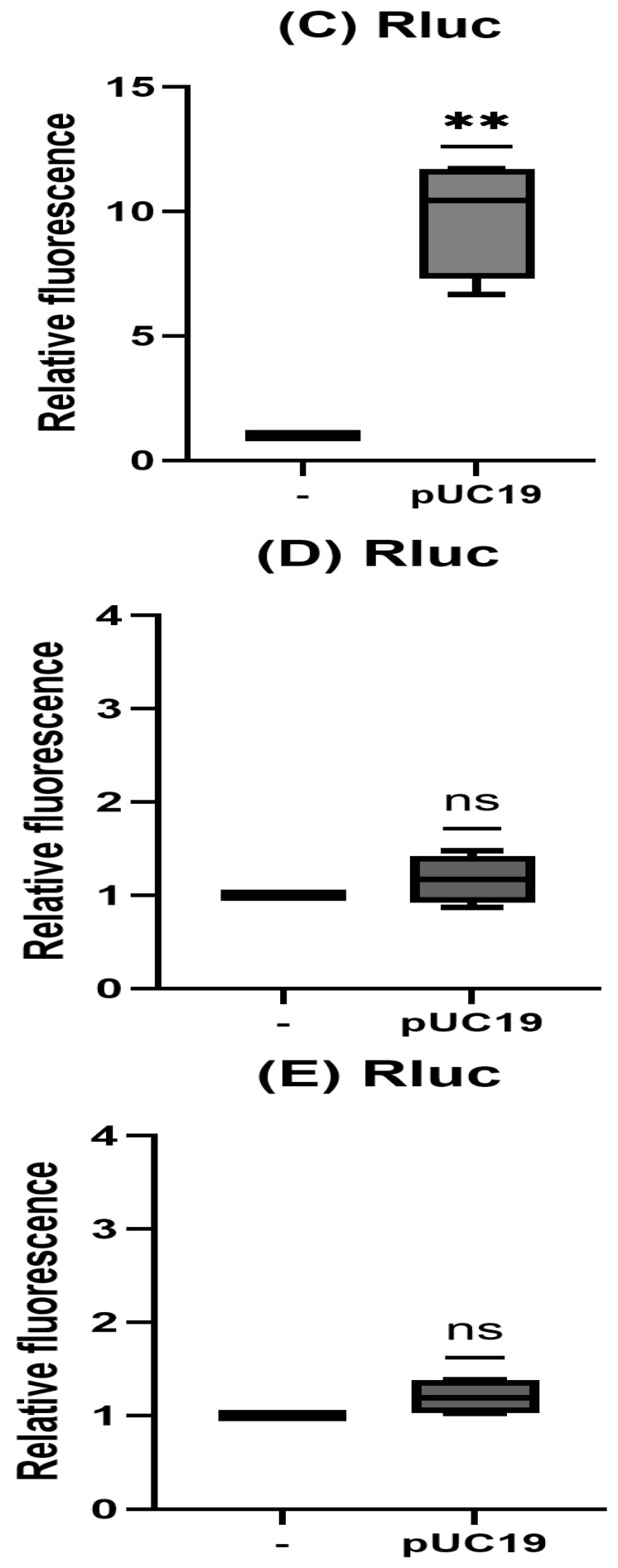

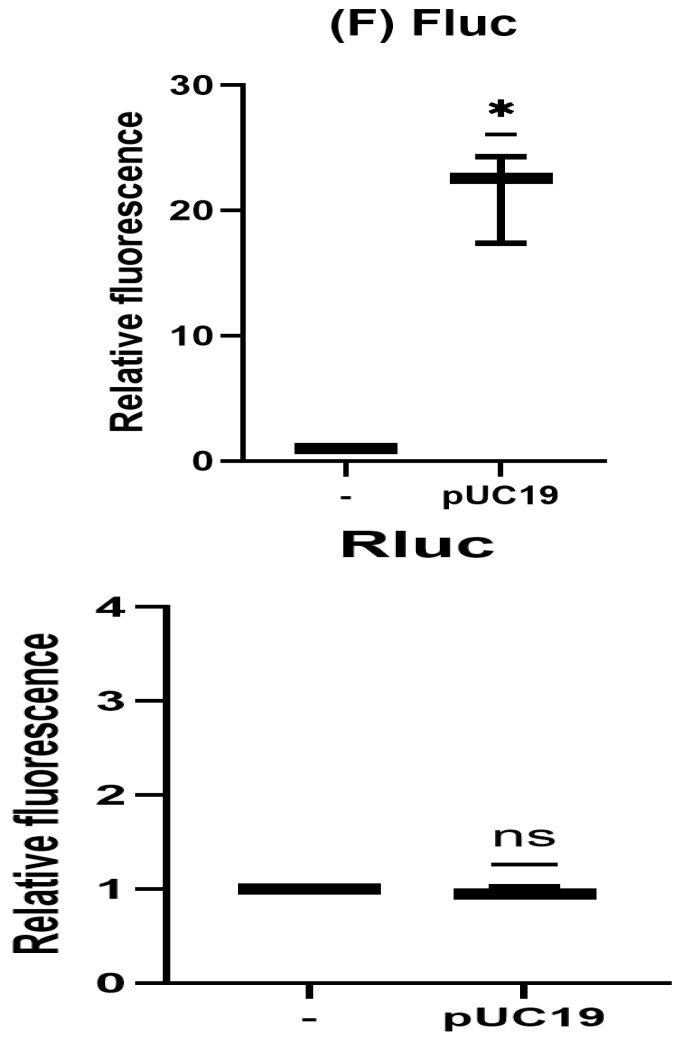
Experiments were performed in triplicate. Luciferase data were normalized to the mock control, which was set to 1. (**A**) HeLa cells stably expressing pLenti-ISRE-Fluc were transfected with different amounts of poly I:C, as indicated. Luciferase activity was analyzed 8 h after transfection. (**B**) HeLa cells were mock-transfected or transfected with 0.4 μg pUC19 in the same amount of PEI reagent. At 4 h after transfection, 0.1 μg pcDNA4-Rluc was transfected. Luciferase activity was analyzed 48 h after transfection. (**C**) HeLa cells were co-transfected with 0.1 μg pcDNA4-Rluc plus 0.4 μg pUC19 or left unmodified in the same amount of PEI reagent. Luciferase activity was analyzed 48 h after transfection. (**D**) HeLa cells were transfected with 0.1 μg pcDNA4-Rluc. After 4 h, cells were mock-transfected or transfected with 0.4 μg pUC19 in the same amount of PEI reagent. Luciferase activity was then analyzed 44 h after transfection. (**E**) HeLa cells were transfected with 0.1 μg pcDNA4-Rluc. At 24 h after transfection, medium of these cells was replaced with the medium from those cells which were mock-transfected or transfected with 0.4 μg pUC19 24 h ago. Luciferase activity was analyzed 24 h later. (**F**) HeLa cells were transfected with (1) 0.4 μg pcDNA4-Fluc, (2) 0.4 μg pcDNA4-Fluc and 1.6 μg pUC19, (3) 0.4 μg pRL-TK. At 6 h after transfection, all of the cells were trypsinized and co-cultured (3) with (1) or (2) in a 1:1 ratio [labeled as—and pUC19]. Luciferase activity was then analyzed 42 h after co-culture. (ns, no significance; *p* < 0.05, *; *p* < 0.01, **; *p* < 0.001, ***).

The results of several previous studies have demonstrated that cytoplasmic dsDNAs can activate the production of interferon in cells [22,23,24,25,26]. HeLa cells stably expressing the reporter Fluc under the control of an ISRE promoter were established through a lentiviral vector (pLenti-ISRE-Fluc), with this reporter being activated by poly I:C (polyinosinic–polycytidylic acid) in a dose-dependent manner (Figure 2A). However, various transfected plasmids (shown in Figure 1A) did not activate this reporter. Moreover, medium from the cells treated with poly I:C but not those from the cells transfected with various plasmids was able to activate the ISRE reporter, with these results indicating that transfected plasmids did not generate detectable interferon production.

pUC19 was able to enhance transient expression of the reporter more significantly in the co-transfection condition than when it was transfected either before or after the reporter (Figure 2B–D). As expected, medium from pUC19-transfected cells did not enhance the transient expression in cells with the reporter (Figure 2E). Moreover, pUC19 only enhances the transient expression of the reporter in the same cells but not in neighboring cells (Figure 2F). These results suggest that the transient expression enhancement of the reporter by the co-transfected plasmids is not induced by the induction of cytokines (e.g., interferon).

### 3.2. Co-Transfected Plasmids Enhance the Transient Transcription of the Reporter Plasmid

**Figure 3:**
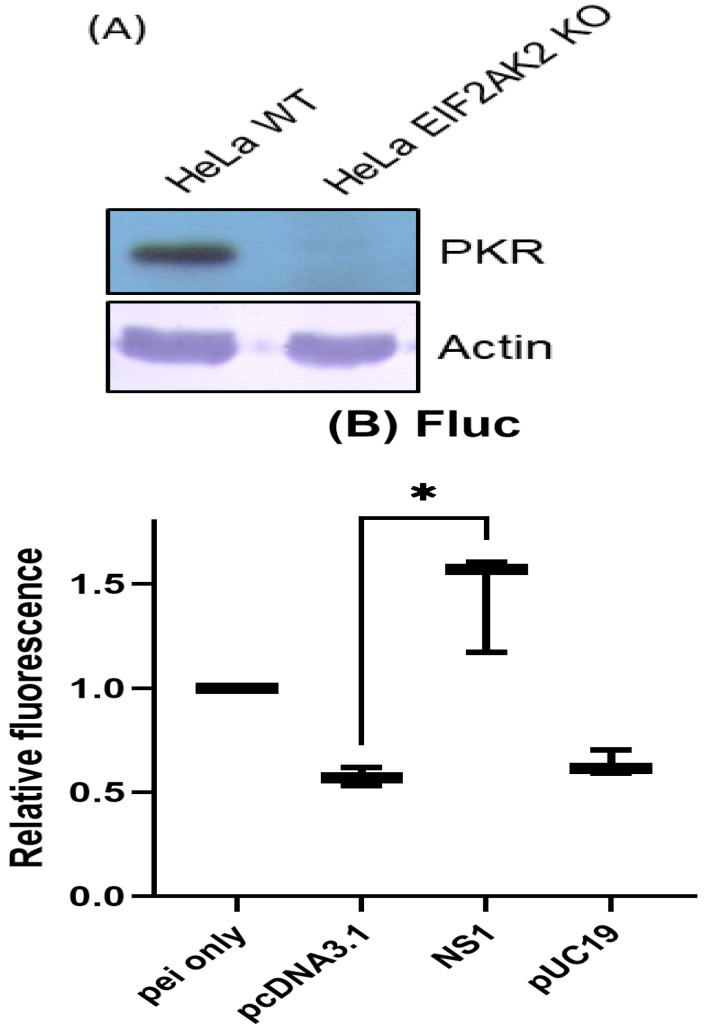

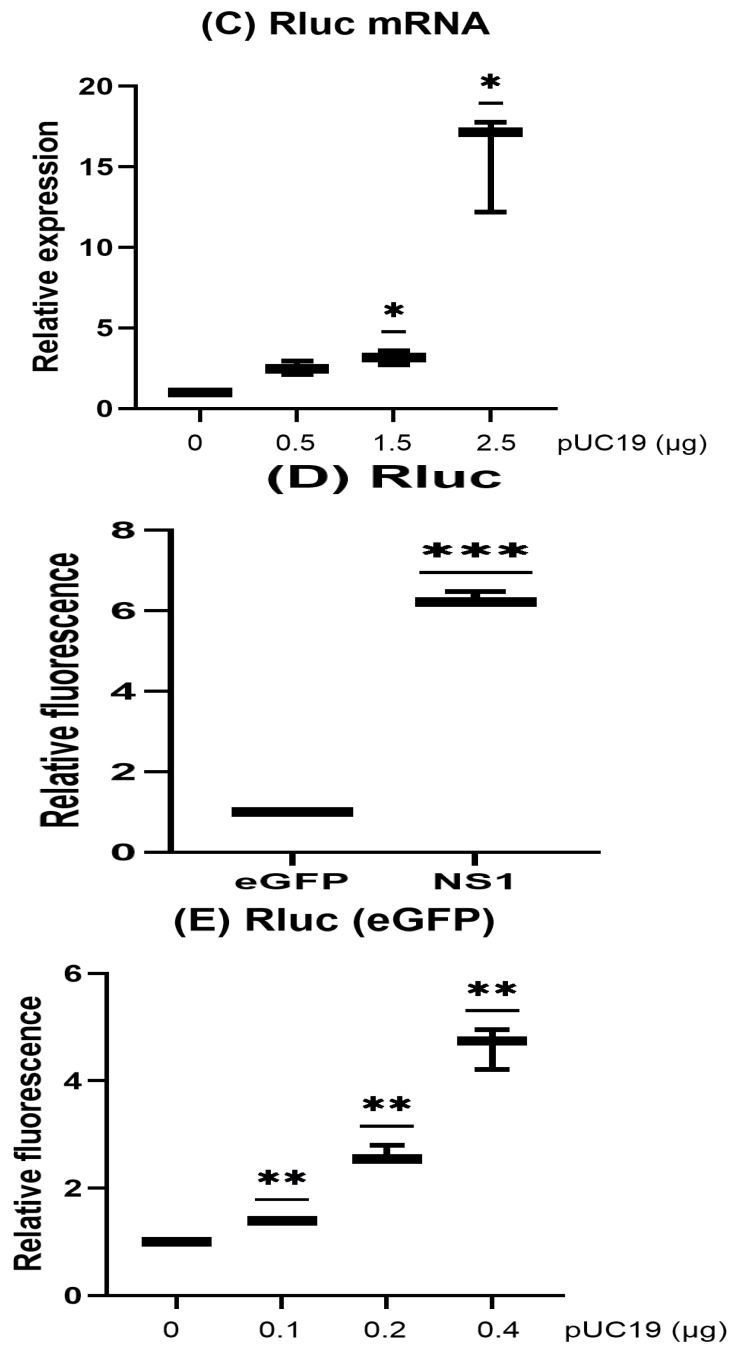

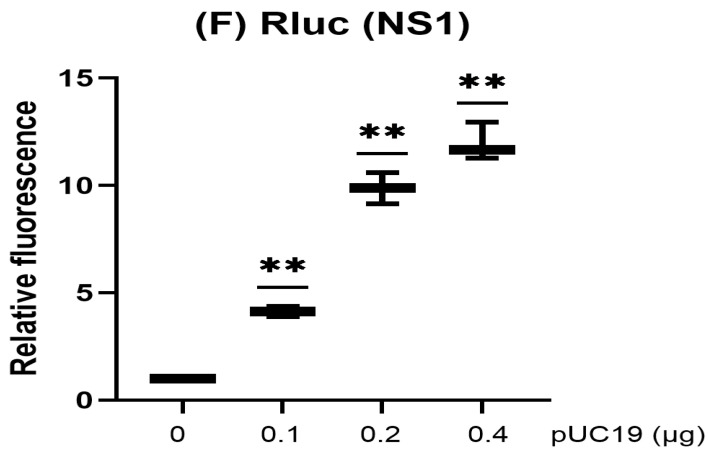
(**A**) Cell lysates from HeLa (WT) and HeLa (eIF2aK2KO) were collected for SDS-PAGE and Western blotting analysis using PKR antibodies as indicated. Actin served as a loading control. (**B**–**F**) Experiments were performed in triplicate. Luciferase data were normalized to the mock control, which was set to 1. (**B**) HeLa (eIF2aK2KO) cells were mock-transfected or transfected with various plasmids as indicated. Then, 24 h after transfection, 0.2 μg CleanCap FLuc mRNAs were transfected. Luciferase activity was then analyzed 24 h after RNA transfection. (**C**) HeLa cells were co-transfected with pcDNA4-Rluc 1 μg and different amounts of pUC19 plasmid as indicated in the same amount of PEI reagent. At 48 h after transfection, RNAs were extracted and analyzed via real-time RT-PCR using b-actin as an internal control. (**D**) HeLa cells stably expressing either eGFP or IAV NS1 were transfected with 0.1 μg pcDNA4-Rluc. Luciferase activity was analyzed 48 h after transfection. (**E**) HeLa cells stably with eGFP were transfected with different amounts of pUC19 plasmid as indicated. Luciferase activity was analyzed 48 h after transfection. (**F**) HeLa cells stably expressing IAV NS1 were transfected with different amounts of pUC19 plasmid as indicated. Luciferase activity was analyzed 48 h after transfection. (*p* < 0.05, *; *p* < 0.01, **; *p* < 0.001, ***).

The interferon-inducible, RNA-dependent protein kinase (PKR) protein is recognized for its ability to inhibit the translation of mRNAs [27,28]. To more effectively analyze the translation of the mRNA reporter, PKR-knockout (eIF2αK2KO) HeLa cells were purchased for analysis (https://www.abcam.com/en-us/search#q=eIF2%CE%B1K2%2C%20hela&sortCriteria=relevance, accessed on 25 November 2025). No PKR protein was detected in these HeLa (eIF2αK2KO) cells (Figure 3A). The commercially available FLuc mRNA expressing a luciferase protein (CleanCap RNA) was used as the reporter. Influenza A virus (IAV) NS1 protein is widely recognized to enhance the translation of the reporter [15,29,30]. To test the effect of transfected plasmids on the translation of the reporter, the pcDNA3.1 vector, pcDNA3.1-NS1, or pUC19 was transfected separately 24 h before FLuc mRNA transfection. Under these conditions, NS1 but not co-transfected plasmids (pcDNA3.1 vector or pUC19) enhances the translation of FLuc mRNA in HeLa (eIF2aK2KO) cells (Figure 3B).

To investigate the effect of transfected plasmids on the transcription of the reporter, the DNA and RNA levels of the reporter pcDNA4-Rluc were analyzed after the reporter pcDNA4-Rluc was co-transfected with different amounts of pUC19 in HeLa cells. While the DNA level of pcDNA4-Rluc was not affected by pUC19, the mRNA level of pcDNA4-Rluc was increased by the pUC19 plasmid in a dose-dependent manner (Figure 3C). These results suggest that co-transfected plasmids may enhance the transient expression of the reporter plasmid at the transcription level.

If pUC19 enhances the transient transcription of the reporter and IAV NS1 protein enhances the translation of the reporter, they should have an additive effect. HeLa cells stably expressing control eGFP and IAV NS1 proteins were established separately [15]. As expected, the transient expression of the reporter pcDNA4-Rluc was enhanced in cells with IAV NS1 proteins, compared to that in cells with eGFP (Figure 3D). When the reporter pcDNA4-Rluc was co-transfected with different doses of pUC19, pUC19 was able to enhance the transient expression of the reporter in both cells dose-dependently (Figure 3E,F). pUC19 was able to enhance transient expression to a greater degree in the cells with NS1 protein than in those with control eGFP (Figure 3E,F). These results suggest that pUC19 and IAV NS1 protein have an additive effect on enhancing the transient expression of the reporter.

### 3.3. Co-Transfected Plasmids Enhance Transient Expression by Competing with Inhibitors

**Figure 4:**
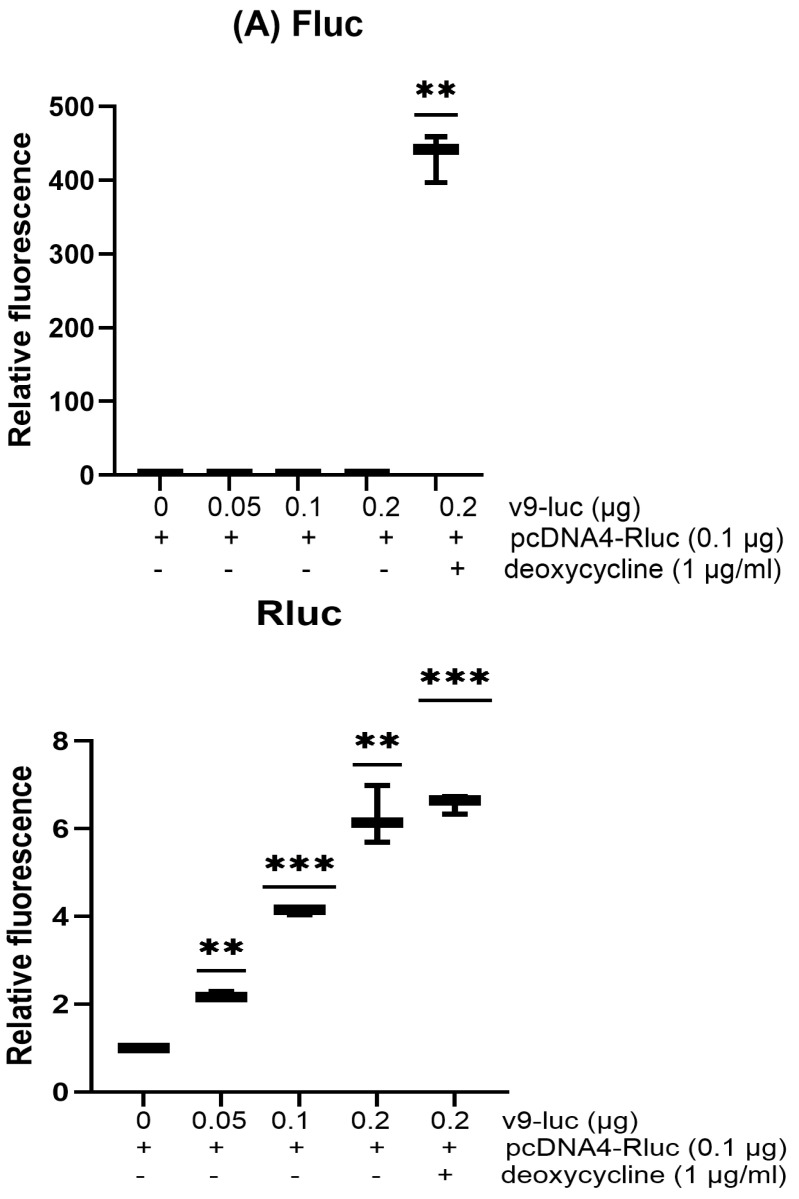

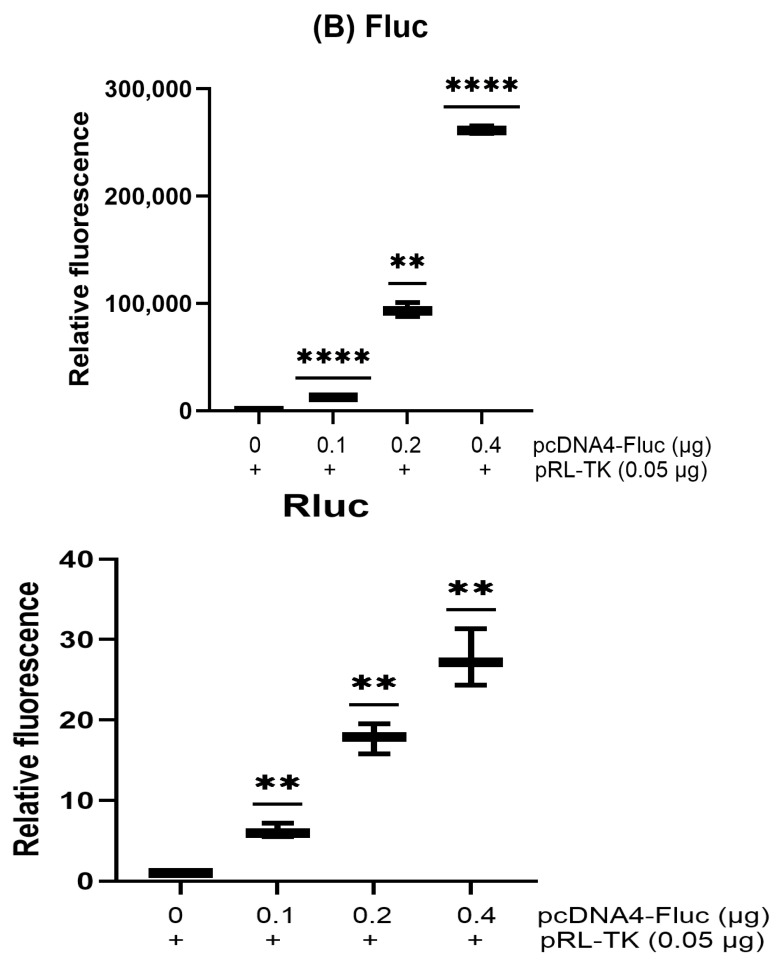

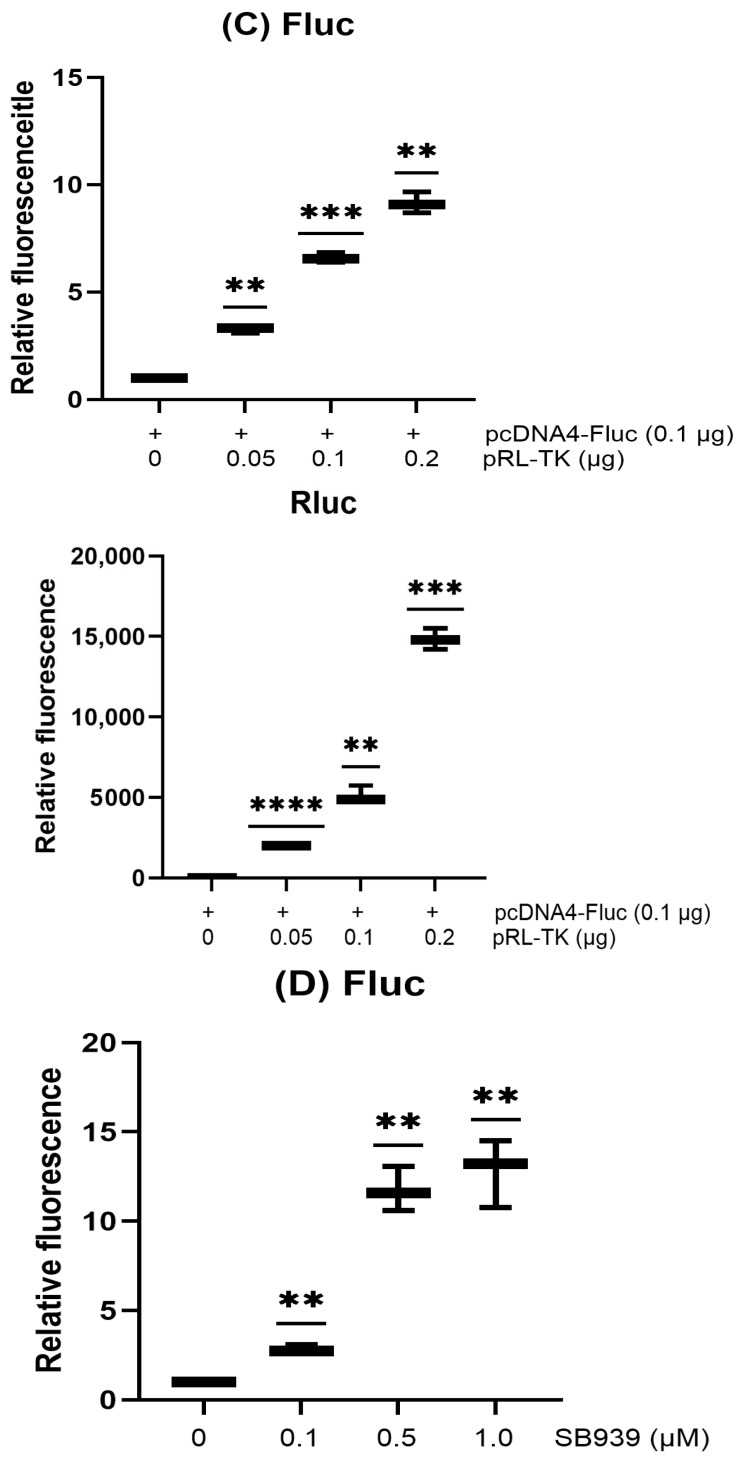

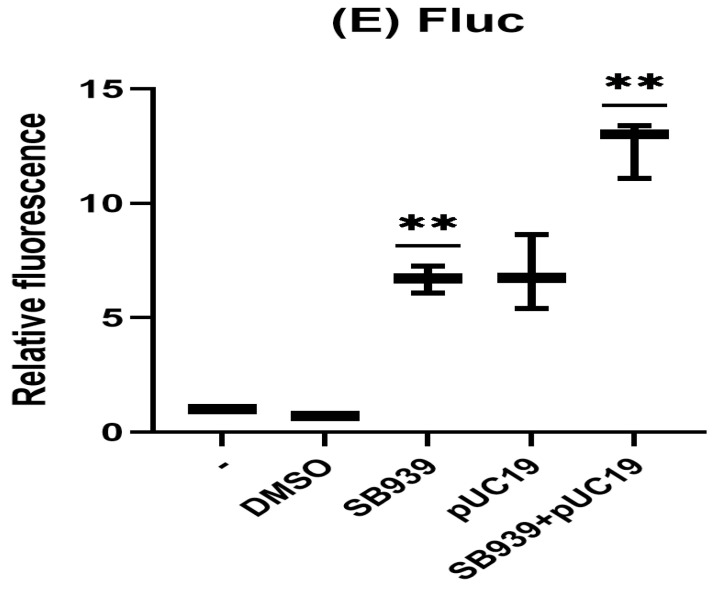
Experiments were performed in triplicate. Luciferase data were normalized to the mock control, which was set to 1. (**A**) HeLa cells were co-transfected with 0.1 μg pcDNA4-Rluc and different amounts of inducible v9-luc as indicated. After transfection, 1 μg/mL of deoxycycline was added to induce Fluc expression. Luciferase activity was analyzed 48 h after transfection. (**B**) HeLa cells were co-transfected with 0.05 μg pRL-TK and different amounts of pcDNA4-Fluc as indicated. Luciferase activity was analyzed 48 h after transfection. (**C**) HeLa cells were co-transfected with 0.1 μg pcDNA4-Fluc and different amounts of pRL-TK as indicated. Luciferase activity was analyzed 48 h after transfection. (**D**) HeLa cells were transfected with pcDNA4-Fluc 0.1 μg and treated with different amounts of SB939 as indicated. Luciferase activity was analyzed 48 h after transfection. (**E**) HeLa cells were transfected with 0.1 μg pcDNA4-Fluc or co-transfected with 0.1 μg pcDNA4-Fluc and 0.2 μg pUC19 plasmid and mock-transfected or treated with 0.5 uM SB939 as indicated. Luciferase activity was analyzed 48 h after transfection. (*p* < 0.01, **; *p* < 0.001, ***; *p* < 0.0001, ****).

To determine whether the expression of the co-transfected plasmids would affect the transient expression of the reporter, HeLa cells were co-transfected with the reporter pcDNA4-Rluc and inducible v9-luc in varying amounts (Figure 4A). As expected, the co-transfected v9-luc plasmid without induction of Fluc protein expression enhanced the transient expression of the reporter pcDNA4-Rluc in a dose-dependent manner. At the same time, inducting the v9-luc plasmid to express Fluc protein did not further affect the transient expression of the reporter pcDNA4-Rluc. Additionally, the co-transfected plasmid pcDNA3-pri-miR122 expressing miRNA122 and pcDNA3.1-actinB expressing cellular actin protein were also able to enhance the transient expression of the reporter, similar to that of pcDNA3.1-V5-HA. These results indicate that the production of mRNA and protein from the co-transfected plasmids did not further modulate the transient expression of the reporter.

To determine whether two co-transfected expressing plasmids could mutually enhance transient expression, HeLa cells were co-transfected with a fixed amount of pRL-TK and varying amounts of pcDNA4-Fluc (Figure 4B). As expected, FLuc activity increased with an increased amount of transfected pcDNA4-Fluc. In this case, Rluc activity also increased with an increased amount of transfected plasmid pcDNA4-Fluc (Figure 4B). Similarly, when HeLa cells were co-transfected with a fixed amount of pcDNA4-Fluc and varying amounts of pRL-TK (Figure 4C), both FLuc and Rluc activities increased with the increasing amount of transfected pRL-TK (Figure 4C). It is unlikely to activate promoter activity through DNA itself. Thus, based on previous results, one possible explanation could be that the enhancement of transient expression of the reporter by the co-transfected plasmid may be the result of competition for factors interacting with transfected DNA to inhibit gene transcription, e.g., histones [31].

Histone-modifying enzymes were previously identified as mediators of polymer-mediated transgene expression from plasmid DNA [32,33]. To determine whether histone-modifying enzymes modulate the transient expression of the transfected plasmids, inhibitors targeting these enzymes were used. When HeLa cells were transfected with reporter pcDNA4-Fluc and treated with Pracinostat (SB939), a small-molecule histone deacetylase (HDAC) inhibitor, reporter activity was enhanced by SB939 dose-dependently (Figure 4D). Enhancement of transient expression of the reporter by SB939 was also detected in HuH7, Vero, and A549 cells. Conversely, tranylcypromine (2-PCPA), an irreversible inhibitor of LSD1 histone demethylase, had no statistically significant effect on the transient expression of the reporter. Moreover, neither ISRIB (integrated stress response inhibitor), an experimental drug that reverses the effects of eIF2α phosphorylation, nor UNC1999, a potent and selective inhibitor of EZH2/1, had a statistically significant effect on the transient expression of the reporter.

To determine the combined effect of pUC19 and SB939 on transient expression, HeLa cells were transfected with reporter pcDNA4-Fluc or co-transfected with the reporter with pUC19 plasmid and mock-treated or treated with SB939 (Figure 4E). Under these conditions, both pUC19 and SB939 were able to enhance transient gene expression, and pUC19 was able to further increase the enhancement of transient reporter expression by SB939 (Figure 4E).

### 3.4. Co-Transfected Plasmids with Different Forms Modulate the Transient Expression of the Reporter Differentially

**Figure 5:**
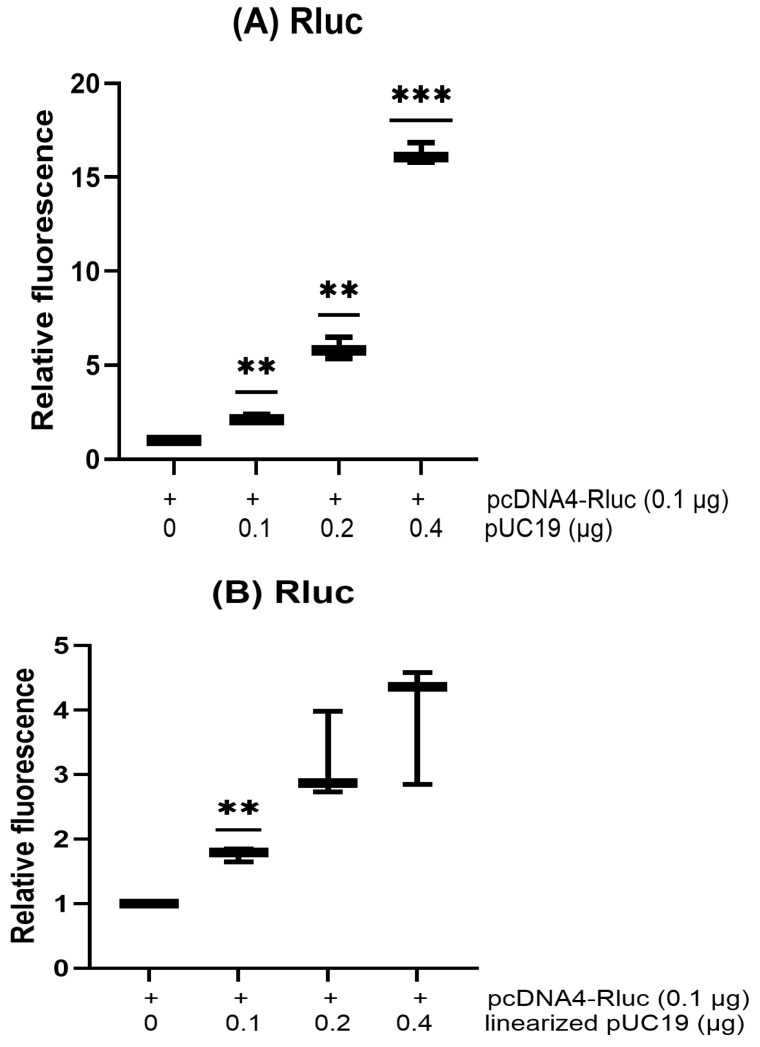

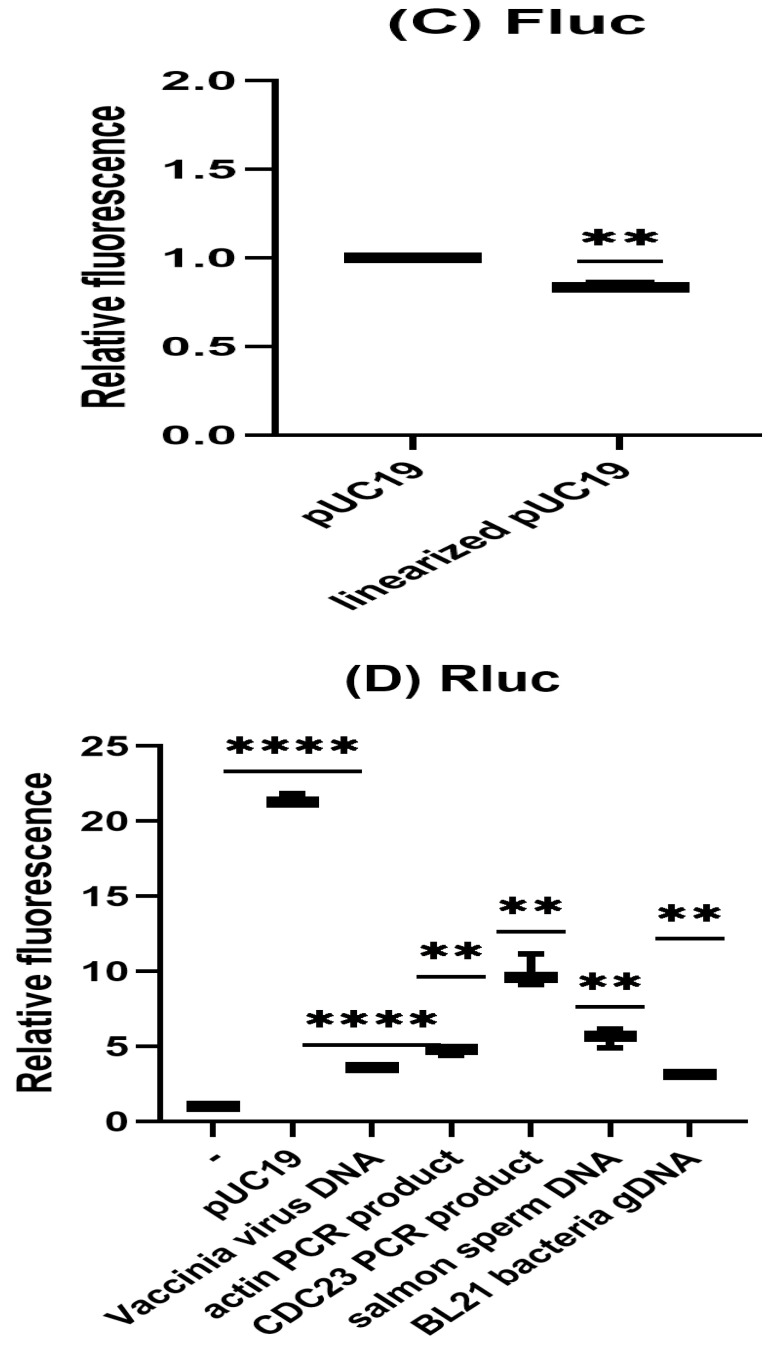
Experiments were performed in triplicate. Luciferase data were normalized to the mock control, which was set to 1. (**A**,**B**) HeLa cells were co-transfected with pcDNA4-Rluc 0.1 μg and different amounts of circular pUC19 plasmid (**A**) or linearized pUC19 (**B**) as indicated in the same amount of PEI reagent. Luciferase activity was analyzed 48 h after transfection. (**C**) HeLa cells stably expressing pLenti-IFN-Fluc were transfected with 0.4 μg of circular pUC19 or linearized pUC19 with EcoRI. Luciferase activity was analyzed 48 h after transfection. (**D**) HeLa cells were co-transfected with 0.1 μg pcDNA4-Rluc and 0.4 μg of various DNA fragments as indicated in the same amount of PEI reagent. Luciferase activity was analyzed 48 h after transfection. (*p* < 0.01, **; *p* < 0.001, ***; *p* < 0.0001, ****).

Histone H1 has been demonstrated to possess a stronger binding preference for supercoiled DNA forms than for linear DNA [34]. If histones are one of the inhibitors interacting with plasmids to suppress the transcription of the reporter, we would expect that co-transfected circular plasmids would enhance the transient expression of the reporter to a greater degree than linear DNAs. To test this hypothesis, HeLa cells were co-transfected with reporter pcDNA4-Rluc and varying amounts of circular pUC19 plasmid (Figure 5A) or pUC19 linearized by restriction enzymes BamHI and XbaI (Figure 5B). As expected, co-transfected circular plasmids enhanced the transient expression of the reporter to a greater degree than linear DNAs. This finding is not due to the induction of interferon production because linear plasmids did not activate the interferon promoter to a greater degree than circular plasmids (Figure 5C).

Linear DNAs with different sizes from different sources, e.g., PCR products (actin, CDC23), vaccinia virus genomes, genomic DNA from E. coli BL21 cells, and sheared salmon sperm DNA [https://www.thermofisher.com/order/catalog/product/tw/en/15632011, accessed on 25 November 2025], were also co-transfected with the reporter to determine their enhancement of the reporter’s transient expression (Figure 5D). None of these DNA fragments were able to enhance the transient expression of the reporter more than circular plasmid pUC19 (Figure 5D).

### 3.5. Co-Transfected Plasmids Only Modulate Pol II Promoters

**Figure 6:**
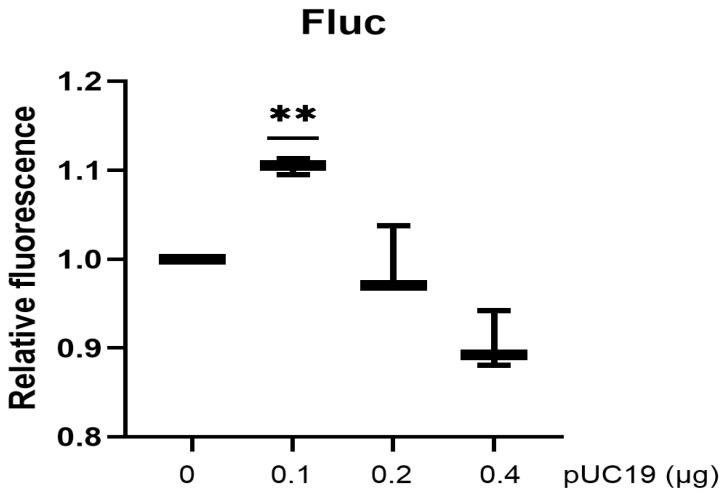
HeLa cells were co-transfected with pU6-Fluc 0.5 μg and different amounts of pUC19 plasmid as indicated in the same amount of PEI reagent. Luciferase activity was analyzed 48 h after transfection. Experiments were performed in triplicate. Luciferase data were normalized to the mock control, which was set to 1. (*p* < 0.01, **).

In addition to the Pol II promoter, the reporters were also constructed under the control of Pol I (pFlu-Rluc) and Pol III (pU6-Fluc) promoters separately. The reporter activity of pU6-Fluc was high [35,36], whereas that of pFlu-Rluc was very low, similar to that of pGL3-basics. When HeLa cells were co-transfected with pU6-Fluc and varying amounts of pUC19 plasmid (Figure 6), pUC19 could not enhance the transient expression of the pU6-Fluc reporter, neither did various co-transfected plasmids, as shown in Figure 1A, enhance the transient expression of pU6-Fluc. Moreover, pUC19 was able to enhance the transient expression of neither pFlu-Rluc nor pGL3-basics. It is thus evident that co-transfected plasmids could only enhance the transient expression of the reporter under the control of the Pol II promoter.

## 4. Discussion

The co-transfected plasmid pUC19 not only enhanced the transient expression of the reporters containing a conventional 5′-untranslated region (5′-UTR), such as pcDNA4-Fuc and pRL-TK, but also enhanced the transient expression of the reporters containing different internal ribosome entry sites (IRESs, from EMCV, HCV, and CRPV), such as pDNA4-EMCV-Rluc, pDNA4-HCVires-Rluc, pDNA4-CRPV-Rluc, both ORFs in pFr-crpv-xb, pcdna4-fluc-emcvires-rluc, pLas2w-fluc-hcvires-rluc, and pLas2w-fluc-crpvigr-rluc. These results suggest that pUC19 may not affect the translation of these reporters because they contain different 5′-UTRs. This hypothesis is supported by the finding that pUC19 did not enhance the translation of in vitro-transcribed CleanCap RNA.

The intracellular DNAs within the cytosol or endosomal compartments are sensed by certain receptors that initiate an innate immune response. Several DNA-sensing molecules, such as DNA Pol III [22], AIM2 [23], IFI16 [24,37,38,39], Cyclic GMP-AMP [25,37,39,40,41,42,43], and DDX41 [26], have been reported to induce cytokine production, e.g., interferon. However, transfected DNAs did not induce interferon production as analyzed via the reporter assay in this study. One possibility is that transfected DNAs were delivered to the nucleus fast enough to avoid the detection of these known DNA sensors. Another possible explanation is that the sensitivity of the reporters used in this study to determine interferon expression and activation (i.e., IFN-luciferase and ISRG-luciferase) is too low. Even if the second hypothesis is true, it does not argue against the conclusion that enhancement of transient expression of the reporter gene by co-transfected plasmids is not initiated through cytokines because this effect is only observed in the same cells but not in neighboring cells (Figure 2). Moreover, enhancement of transient expression of the reporter gene by co-transfected plasmids is also detected in Vero cells, which lack interferon genes [44,45].

In this study, co-transfected plasmids were found to markedly enhance the transient expression of the reporter gene in various cells, possibly through competition between the co-transfected plasmids and reporter plasmids for cellular proteins that interact with transfected DNAs, such as histones. Transient DNA transfection is routinely used to study the regulation of gene function. The results of this study echo those of previous studies in that competition for cellular resources during co-transfection experiments can bias data interpretation. Transient DNA transfection is often used in biotechnology for large-scale recombinant protein production. The findings presented in this study should also provide strategies to facilitate transient expression of recombinant genes in biotechnological applications, such as the inhibition of histones.

Our results, demonstrating that pUC19 could further enhance SB939-induced transient reporter gene expression (Figure 4E), suggest that there are other DNA-binding molecules, in addition to histones, inhibiting the transient expression of the reporters. Several proteins have been reported to interact with transfected DNAs, such as DNA Pol III [22,39,40,41], AIM2 [23], IFI16 [24], Cyclic GMP-AMP [25], and DDX41 [26]. Whether these proteins are involved in inhibiting the transient expression of the transfected plasmids is under investigation. In addition to the saturation of innate DNA sensors by the co-transfected plasmids, there are alternative or additional mechanisms such as enhancement of the nuclear import of the reporters by the co-transfected plasmids or reduction in the non-specific chromatin effects of the reporters by the co-transfected plasmids.

The main limitation of the current study is the lack of direct assays, such as the chromatin immunoprecipitation assay for histone occupancy on the reporter plasmid, or direct comparison of histone binding in the presence or absence of co-transfected plasmid DNA, to support the proposed mechanism regarding the interactions between the transfected DNAs and cellular proteins, such as histones and/or DNA sensors.

## 5. Conclusions

Transient DNA transfection is used in the field of biotechnology for large-scale recombinant protein production. In this study, we found that co-transfected plasmids markedly enhance transient—but not stable—expression of various reporter genes across different cell types. This study thus provides insights that may facilitate improved transient expression of recombinant genes in biotechnological applications.

## Figures and Tables

**Table 1:** Description of the reporters used in this study.

Characteristics
pLAS5w-Fluc: pLAS5w-pPuro vector expressing Firefly luciferase (Fluc) under the control of the elongation factor 1 alpha promoter
pRL-TK: Renilla luciferase expression under the control of the thymidine kinase promoter
pcDNA4-Fluc: pcDNA4 vector expressing Firefly luciferase under the control of the CMV promoter
pcDNA4-Rluc: pcDNA4 vector expressing Renilla luciferase (Rluc)
pDNA4-EMCV-Rluc: pcDNA4 vector expressing Renilla luciferase with EMCV IRES as the 5′-UTR
pDNA4-HCVires-Rluc: pcDNA4 vector expressing Renilla luciferase with HCV IRES as the 5′-UTR
pDNA4-CRPV-Rluc: pcDNA4 vector expressing Renilla luciferase with CrPV IRES as the 5′-UTR
pfr-crpv-xb: pcDNA4 vector expressing Fluc and Rluc with the insertion of CrPV IRES
pcdna4-fluc-emcvires-rluc: pcDNA4 vector expressing Fluc and Rluc with the insertion of EMCV IRES
pLAS2w-fluc-hcvires-rluc: pLAS2w vector expressing Fluc and Rluc with the insertion of HCV IRES
pLAS2w-fluc-crpvigr-rluc: pLAS2w vector expressing Fluc and Rluc with the insertion of CrPV IRES
pGL3-SERPINE1-p700: Fluc expression under the control of cellular SERPINE1 promoter
v9-luc: Fluc expression under the control of an inducible promoter

Note: Internal ribosomal entry site (IRES) [11]; encephalomyocarditis virus (EMCV) [12]; hepatitis C virus (HCV) [13]; Cricket Paralysis Virus (CrPV) [14].

## Data Availability

The original contributions presented in this study are included in the article. Further inquiries can be directed to the corresponding author.
